# Estimates of prevalence of anti-SARS-CoV-2 antibodies among blood donors in South Africa in March 2022

**DOI:** 10.21203/rs.3.rs-1687679/v2

**Published:** 2022-05-26

**Authors:** Jeremy Bingham, Russel Cable, Charl Coleman, Tanya Nadia Glatt, Eduard Grebe, Laurette Mhlanga, Cynthia Nyano, Nadia Pieterson, Ronel Swanevelder, Avril Swarts, Wendy Sykes, Karin van den Berg, Marion Vermeulen, Alex Welte

**Affiliations:** South African DST-NRF Centre of Excellence in Epidemiological Modelling and Analysis, Stellenbosch University; Western Cape Blood Service; South African National Blood Service; South African National Blood Service; Vitalant Research Institute; Northwestern University; South African National Blood Service; Western Cape Blood Service; South African National Blood Service; South African National Blood Service; South African National Blood Service; South African National Blood Service; South African National Blood Service; DSI-NRF Centre of Excellence in Epidemiological Modelling and Analysis

**Keywords:** COVID-19, seroprevalence, anti-SARS-CoV-2 antibodies, blood donors, South Africa

## Abstract

In line with previous instalments of analysis from this ongoing study to monitor ‘Covid Seroprevalence’ among blood donors in South Africa, we report on an analysis of 3395 samples obtained in mid-March 2022 from all provinces of South Africa – a timepoint just after the fourth (primarily omicron) wave of infections. As in our previous analyses, we see no evidence of age and sex dependence of prevalence, but significant variation by race. Differences between provinces have largely disappeared, as prevalence appears to have saturated. In contrast to previous estimates from this study, which reported only prevalence of anti-nucleocapsid antibodies, this present work also reports results from testing for anti-spike antibodies. This addition allows us to categorise those donors whose only antibodies are from vaccination. Our race-weighted national extrapolation is that 98% of South Africans have some antibodies, noting that 10% have anti-spike antibodies but not anti-nucleocapsid antibodies - a reasonable proxy for having both 1) been vaccinated and 2) avoided infection.

## Introduction

We have previously published estimates of the prevalence of anti-SARS-CoV-2 antibodies among blood donors in South Africa, based on specimens collected from January to November 2021 [1,2], as well as estimates of fatality rates [3], based on these prevalence estimates and publicly available excess deaths estimates.

While the interpretation of seroprevalence is increasingly complicated by

- vaccination coverage
- multiple infections of individuals and
- waning of antibody titre to below detectable levels,

we believe that seroprevalence is still relevant from the point of view of understanding aspects of transmission and collective immunity that are relevant to ongoing adjustment of anti-transmission measures, policies, and regulation.

## Methods

As soon as feasible after the omicron variant driven fourth wave in early 2022, 3395 residual specimens were randomly collected from consenting donors from all provinces presenting to donate blood at either of the South African Blood Services: the South African National Blood Service (SANBS), and the Western Cape Blood Service (WCBS) in mid-March 2022. This was carried out in accordance with previous arrangements and practices underlying our previous rounds of sampling [1,2,3], as approved by the SANBS Human Research Ethics Committee (HREC).

For statistical analysis, serology data was linked to basic donor demographic information (age, sex and race) but not to any other underlying data potentially available from the SANBS donor database (donation history, specific locale of donation/residence, donor identifiers etc).

All samples were tested for the presence of antibodies to SARS-CoV-2 nucleocapsid proteins (Roche Elecsys Anti-SARS-CoV-2) and, separately, for the presence of antibodies to SARS-CoV-2 spike proteins (Roche Elecsys AntiSARS-CoV-2 S). Antibodies to the nucleocapsid proteins are typically developed in response to natural infection, but not in response to any vaccine currently available in South Africa. Antibodies to the spike protein are typically developed either from a natural infection or through vaccination alone. While there is some waning of antibodies, in this context we expect most donors who ever developed antibodies to have detectable levels at the time of testing [4] while a small proportion (we estimate in the range of 5–10 percent) of individuals who experience infection remain sero-silent i.e. never develop detectable antibodies. [5]

## Results

**Figure 1:**
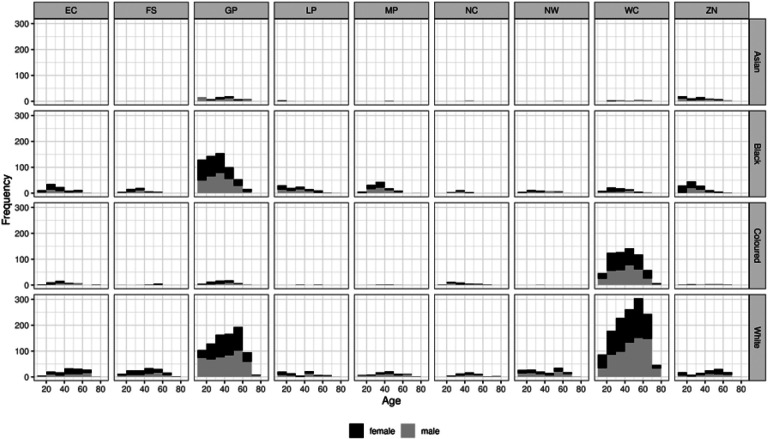
Distribution of specimens obtained, by age and sex, for each province

**Figure 2:**
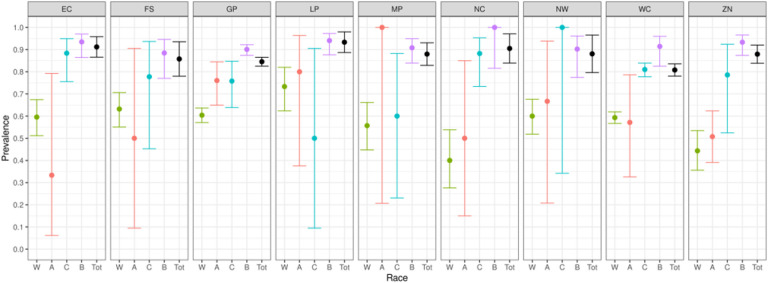
Prevalence of anti-nucleocapsid antibodies

**Figure 3:**
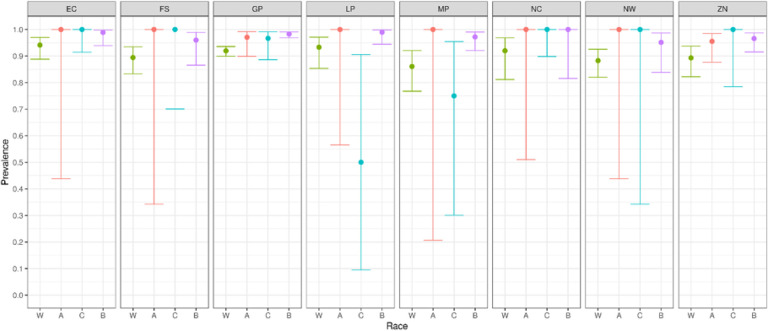
Prevalence of anti-spike antibodies

**Figure 4:**
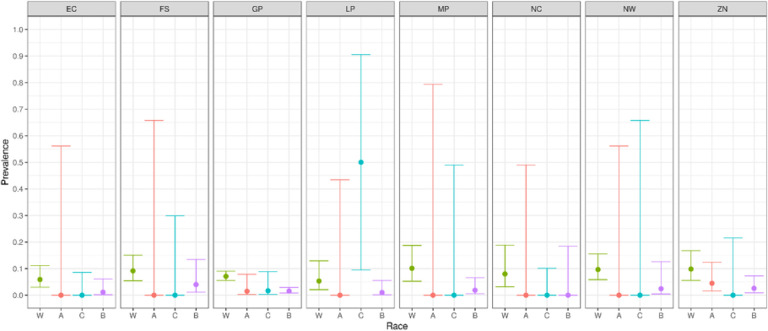
Proportion of donors lacking any antibodies

**Figure 5:**
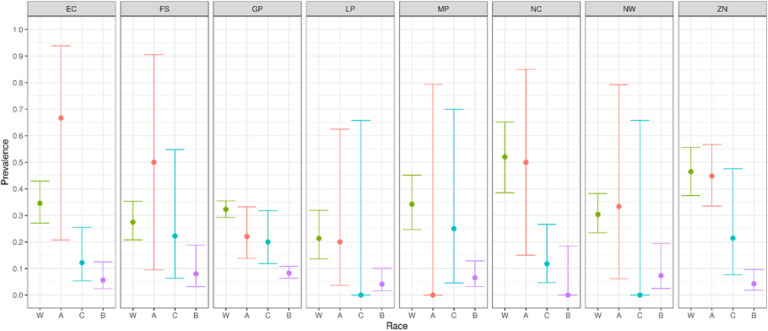
Proportion of donors having anti-spike antibodies, but lacking anti-nucleocapsid antibodies

Figure 1 shows the distribution of specimens by age group, race, sex and province. We found no statistically significant dependence of seroprevalence on either age or sex. However, unsurprisingly, there were statistically and epidemiologically significant differences between race groups. Hence:

- We report primary results by every combination of province and racial group (Figure 2–5).
- From these, we generated race-weighted seroprevalence for each province (Figure 2–5).
- From these provincial estimates, we generated a ‘national’ estimate by weighting according to provincial population sizes (Table 1).

The weighted national prevalence of anti-nucleocapsid antibodies (evidence of infection) is 87% and an additional 10% had only anti-spike antibodies (suggesting vaccination and the lack of natural infection)

## Discussion

As ever, we note well known caveats about representativeness of donors, despite which we believe that our donor population probably does not differ greatly from the general population – especially as the usual reservation is likely to be that donor infection exposure is prone to being an underestimate of population level exposure, and the prevalence estimates are so high in our case.

At this stage of the epidemic there is an increasing incidence of reinfection. In fact, one of the notable features of the omicron wave was the early detection of large numbers of reinfections. Our previous analyses noted a modest increase in seroprevalence during the delta wave – but the present analysis, when compared to our previous estimates from after the delta wave but before the omicron wave, suggests that the infectious pressure of the omicron variant was extraordinarily high, to have produced such a significant bump in prevalence at this relatively mature stage of the epidemic. It is hardly possible to imagine much higher prevalence values, given that there is antibody waning, and not all individuals develop, upon infection, antibodies detectable by any given assay.

It is tempting, if fraught, to interpret the high proportion of white donors who have only anti-spike antibodies. This is a reasonable proxy for the state of “having been vaccinated AND not having been infected”. In this case, the proportions are surprisingly (to us) high – certainly higher than (we) expected from assuming that the vaccination rate among donors is much like in the general population, and that, though the vaccine has high efficacy against severe illness, there is limited efficacy against mild infection. It seems plausible to suggest that white donors are both unusually likely to avail themselves of vaccination, AND they are unusually able to avoid exposure, for instance by working predominantly from home, living in smaller family units, etc.

## Figures and Tables

**Table 1:** National estimates for various key combinations of anti-spike and anti-core antibody positivity

Antibody Type(s)	National Prevalence (%)	95% confidence Interval (%)
anti-nucleocapsid only^1^	0.5	0.1 – 0.9
anti-spike only^2^	10.8	9.4 – 12.1
anti-nucleocapsid and anti-spike^3^	86.5	85.0 – 88.0
anti-spike^4^	97.2	96.5 – 98.0
anti-nucleocapsid^5^	87.0	85.5 – 88.4
Neither^6^	2.2	1.6 – 2.9

Notes:

1. Exhibiting only anti-nucleocapsid antibodies suggests either that both were once present and anti-spike antibodies waned more quickly, or a false positive.

2. Having only anti-spike antibodies is suggestive of vaccination, in the absence of (recent) infection.

3. This is the expected result for someone who has had natural infection not too long ago.

4. Donors exhibiting anti-spike antibodies, with or without anti-nucleocapsid antibodies, is the proxy for exposure to at least either vaccination or natural infection

5. Donors exhibiting anti-nucleocapsid antibodies, with or without anti-spike antibodies, is expected from anyone having had natural infection, whether or not the anti-spike antibody titre has waned or failed to develop to detectable levels

6. Donors will have no antibodies if a) they have never had exposure either to infection or vaccination, or b) have had one or other exposure, but either i) failed to develop detectable levels or ii) have experienced waning titres.
